# Correction: Synthetic 2-D lead tin sulfide nanosheets with tuneable optoelectronic properties from a potentially scalable reaction pathway

**DOI:** 10.1039/c9sc90038a

**Published:** 2019-02-18

**Authors:** Kane Norton, Jens Kunstmann, Lu Ping, Alexander Rakowski, Chuchen Wang, Alexander J. Marsden, Ghulam Murtaza, Niting Zeng, Simon G. McAdams, Mark A. Bissett, Sarah J. Haigh, Brian Derby, Gotthard Seifert, Jack Chun-Ren Ke, David J. Lewis

**Affiliations:** a School of Materials University of Manchester , Oxford Road , Manchester , M13 9PL , UK . Email: david.lewis-4@manchester.ac.uk; b Theoretische Chemie , Technische Universität Dresden , 01069 Dresden , Germany . Email: jens.kunstmann@tu-dresden.de; c School of Chemistry , University of Manchester , Oxford Road , Manchester , M13 9PL , UK; d National Graphene Institute , University of Manchester , Oxford Road , Manchester , M13 9PL , UK; e Photon Science Institute , University of Manchester , Oxford Road , Manchester , M13 9PL , UK

## Abstract

Correction for ‘Synthetic 2-D lead tin sulfide nanosheets with tuneable optoelectronic properties from a potentially scalable reaction pathway’ by Kane Norton *et al.*, *Chem. Sci.*, 2019, **10**, 1035–1045.



## 

In the original manuscript, Jack Chun-Ren Ke was accidentally omitted from the author list. Jack Chun-Ren Ke assisted with this research through the conduction of Raman spectroscopy experiments. In addition, the name of Simon G. McAdams was displayed incorrectly in the original manuscript. The corrected list above demonstrates how the author names should be displayed for this article.

The Royal Society of Chemistry apologises for these errors and any consequent inconvenience to authors and readers.

